# Supplementary material for: High-Throughput Griess Assay of Nitrite and Nitrate in Plasma and Red Blood Cells for Human Physiology Studies under Extreme Conditions
Source: Molecules. 2021 Jul 28;26(15):4569. doi: 10.3390/molecules26154569 (PMC8348835; doi:10.3390/molecules26154569)
Supplement: Supplementary file 1 [file molecules-26-04569-s001.zip › molecules-1289232-supplementary.pdf]

High-throughput Griess assay of nitrite and nitrate in plasma and red blood cells for human physiology studies under extreme conditions

Andrea Brizzolari, Michele Dei Cas, Danilo Cialoni, Alessandro Marroni, Camillo Morano, Michele Samaja, Rita Paroni, Federico Maria Rubino.

molecules-1289232 (2021)

Supplementary Material S1. Operative checklist for the measurement of nitrate and nitrate in human plasma and red blood cells.

General information.

1 Safety notes.

Equipment. All equipment that involves occupational hazard should be in proper operating conditions. Electrical appliances should conform to local regulations and be properly plugged in the sockets to avoid electrical shock and mechanical hurt. Pipetting and other liquid transfer should be performed with properly liquid-tight hardware and disposable plasticware to avoid unnecessary dispersion of reagents and biological samples.

Reagents. All reagents used in this method have been selected for minimal toxicity and absence of carcinogenic activity, as far as current toxicological knowledge allows. Organic solvent and their mixtures with water and with samples are not flammable. Acidic and caustic reagents should be prepared in the minimum amount and handled with proper precautions.

Samples. Trained medical personnel only, wearing appropriate protections, should perform blood sample withdrawal from subjects, under appropriate safety conditions. Biological samples should be handled in the laboratory with due precaution as referring to their origin and possible hazards. After protein precipitation, biological risk is mitigated.

Personal protective equipment. Perform blood fractionation and analysis when wearing the necessary laboratory attire and personal protective equipment. Latex or nitrile disposable gloves, face mask and shield should be worn during the entire procedure. Caution should be exercised to avoid contact of gloved hands with unprotected computer keyboards.

Waste management. The method has been developed to minimize the use of disposable plasticware by planning the number of samples for each lot of analyses. Reagents should be prepared in the appropriate minimum quantities to avoid waste. Spent sample plates should be discarded according to local regulations.

2 General equipment.

a) Cold storage and transport facilities (-+4°C; -20°C).
b) Clinical hemocromocytometer to measure hematological parameters in fresh blood samples.
c) Clinical centrifuge to separate plasma from red blood cells.
d) Mini-centrifuge to separate surnatant from protein precipitate in Eppendorf plastic tubes.
e) Laboratory thermostat for use with polystyrene plates for heating at 55°C.
f) Multiple (8-channel) pipettor for 200 μL disposable tips.
g) Single-channel pipettors with disposable tips: 5,000, 1,000 and 200 μL
h) Plate reader for spectrophotometric measurements at 450 and 540nm. The employed model uses optical filters at 450 nm (filter bandpass 8.5-16 nm) for hemoglobin measurement and at 540 nm (550 nm filter bandpass 10-14 nm) for the measurement of the Griess reaction product.
i) Computer for data storage and elaboration with spreadsheet.

3 General glass and plasticware.

Glassware

Glass 25-mL volumetric flasks for stock solution preparations.

Disposable glassware

Vacutainer-type tubes for blood sampling

Disposable plasticware

Eppendorf-type tubes for in-field blood fractionation with snap-cap or screw-cap (2-mL, 0.5 mL)
Polypropylene tubes with stopcocks (10-mL)
Pipettor disposable tips: 5,000, 1,000 and 200 μL
Reagent reservoirs for multichannel pipettors, 25 mL
Polystyrene reading plates, 96 wells, 250 μL well volume, with lid
Tight-close plastic sample bags
Sanitary box for biological waste disposal
Sanitary box for sharps disposal

4 Reagents.

Hydrochloric acid (1M and 2M solutions from concentrated 37% acid)
Phosphoric acid (85%, 5% v/v in water)
Deionized water from MilliQ system
Acetonitrile, chromatography grade (pure and 2:1 v/v mixture with water)
Sodium nitrite, Sigma S2252
Sodium nitrate, Sigma S8170
Sulphanylamide (SA), Sigma S9251
N-(1-naphthyl)ethylenediamine (NED), Sigma 33461
Vanadyl(III) chloride, Sigma 208272

Procedure for the fractionation of whole blood samples.

a) Blood samples (approx. 3 mL) are obtained by venipuncture in 5-mL vacutainer-type tubes.
b) Rapid cooling to sub-ambient temperature can be achieved in a non-freezing ice-water mixture if samples stand for more than one hour before fractionation. Complete blood fractionation should be performed the soonest possible.
c) From the cooled whole blood sample withdraw a 200- μL whole blood sample in a 0.5 mL Eppendorf cone for hematocrit measurement. Store at +4°C and perform measurement within 24-36 h.
d) Centrifuge the remaining whole blood sample (12,000 rpm, 10 min, +4°C) to separate plasma and red blood cells.
e) Separate plasma in appropriate volume aliquots as follows, without disturbing the settled red blood cells.
    a. 2 x 0.5 mL in 1.5-mL Eppendorf tubes
    b. 1 x remaining plasma volume in one 2.0-mL Eppendorf tube
f) Separate the red blood cells as following.
    a. 4 x 50 μL aliquots of red blood cells in 0.5-mL Eppendorf tubes
    b. 1 x remaining red blood cells in one 2.0-mL Eppendorf tube
g) Place sampled aliquots and reference leaflet in plastic bag for fast cooling at -20°C.

Discard waste in the appropriate containers.

Operative checklist for the measurement of nitrate and nitrate in plasma and red blood cells.

Preparation of standards and reagents.

Preparation of standards.

Sodium nitrite. Accurately weight approx. 70 mg in a 25-mL volumetric flask and dilute with deionized water to a final concentration 40 mM. Dilute appropriate volumes to 200, 100, 50, 20, 10 and 5 microM with a mixture of de-ionized water and acetonitrile in a 1:2 volume ratio. The dilution table is as follows.

**Stock solution in water**			
**Molecular mass $NaNO_3$**	69,0	**mg / mmole**	
**Weight (mg) in 25 mL**	71,2	1,03 mmole	
**Concentration of stock**	**mmole/(25 mL/1000)**	41.28 mM	
**Working Std in water**	1 mM =	0.242 mL/10 mL H_2O	1,00 mM
**Calibrator microM (target)**	mL WkSt/10mL	**microM (actual)**	in
200	2.000	200,2	H_2O/MeCN 1:2 v/v
100	1.000	100,1	
50	0.500	50,0	
20	0.250	20,0	
10	0.100	10,0	
5	0.050	5,0	
0	0	0,0	

Sodium nitrate. Accurately weight approx. 90 mg in a 25-mL volumetric flask and and dilute with deionized water to a final concentration 40 mM. Dilute appropriate volumes to 200, 100, 50, 20, 10 and 5 microM with a mixture of de-ionized water and acetonitrile in a 1:2 volume ratio. The dilution table is as follows.

**Stock solution in water**			
**Molecular mass $NaNO_3$**	84.99	**mg / mmole**	
**Weight (mg) in 25 mL**	90.1	1,06 mmole	
**Concentration of stock**	**mmole/(25 mL/1000)**	42.40 mM	
**Working Std in water**	1 mM =	0.237 mL/10 mL H_2O	1,00 mM
**Calibrator microM (target)**	mL WkSt/10mL	**microM (actual)**	in
200	2.000	200,2	H_2O/MeCN 1:2 v/v
100	1.000	100,1	
50	0.500	50,0	
20	0.250	20,0	
10	0.100	10,0	
5	0.050	5,0	
0	0	0,0	

Hemoglobin. Accurately weight an approx. 2.5 mg sample in a 2-mL Eppendorf tube and dilute with deionized water to a final concentration 40 microM. Dilute appropriate volumes to 20, 10, and 5 microM (e.g., 0.5 mL solution + 0.5 mL water) with deionized water for spectrophotometric measurement. The dilution table is as follows.

a) Weight of human Hemoglobin standard: 2.5 mg
b) Amount of human Hemoglobin (micromoles): 10^6 * Weight / 64.454 = 38.78
c) Volume of water (mL) to obtain a 40 microM Hb solution: 40 / (b) = 0.970

Take 0.5 mL of solution (c) and dilute with 0.5 mL of water (solution (d), 20 microM)
Take 0.5 mL of solution (d) and dilute with 0.5 mL of water (solution (e), 10 microM)
Take 0.5 mL of solution(e) and dilute with 0.5 mL of water (solution (f), 5 microM)

Use solutions (c) – (f) for the standard curve of Plate C (see below)

Preparation of reagents.

Vanadyl chloride. For the batch of 96 samples, weight approx. 40 mg VCl_3 (MW 157.30) in a 10-mL stoppered plastic tube and dilute to a final concentration 25 mM in 1 M hydrochloric acid.
Sulphanylamide (Griess reagent A). For the batch of 96 samples, weight approx. 150 mg (MW 172.20) in a 10-mL stoppered plastic tube and diluted to a final concentration 87 mM in 2M hydrochloric acid.
N-(1-naphthyl)ethylenediamine (Griess reagent B). For the batch of 96 samples, weight approx. 10 mg (MW 259.18) in a 10-mL plastic tube and dilute to a final concentration of 3.9 mM in 5% v/v phosphoric acid.
Combined Griess reagent. For the batch of 96 samples, 10 mL of reagent A and 10 mL of reagent B are combined shortly before sample preparation.

Operative checklist for the measurement of nitrite and nitrate in plasma.

Protein precipitation

Procedure for plasma or serum. From each thawed and mixed plasma sample, dispense 100 μL into a 1.5 mL Eppendorf-type centrifuge tube. To each tube, add 200 μL of chilled (-20°C) acetonitrile and vortex the closed tube immediately for 5 sec, then centrifuge each lot of 6 samples at 12,000 rpm for 10 min. withdraw from the tube two 100-μL subsamples for separate nitrite and nitrate measurement.

Sample plate preparation and measurement

According to the number of measured samples, one plate per measurement is prepared, with samples in matching positions. The sample loading scheme below is presented for the analysis of a 80-sample batch in 96-well plates.

Fresh reagent batches were prepared from the solids and used immediately after.

Plate A: measurement of nitrite

	1	2	3	4	5	6	7	8	9	10	11	12
**A**	○0	○0	○1	○2	○3	○4	○5	○6	○7	○8	○9	○10
**B**	○5	○5	○11	○12	○13	○14	○15	○16	○17	○18	○19	○20
**C**	○10	○10	○21	○22	○23	○24	○25	○26	○27	○28	○29	○30
**D**	○20	○20	○31	○32	○33	○34	○35	○36	○37	○38	○39	○40
**E**	○50	○50	○41	○42	○43	○44	○45	○46	○47	○48	○49	○50
**F**	○100	○100	○51	○52	○53	○54	○55	○56	○57	○58	○59	○60
**G**	○200	○200	○61	○62	○63	○64	○65	○66	○67	○68	○69	○70
**H**	○v	○v	○71	○72	○73	○74	○75	○76	○77	○78	○79	○80

Legend.

○0-200: NO_2^- calibrator (μM)	○n: sample (1-80)	

In the 96-well plate, for each well, the following volumes of sample and reagents are dispensed.

a) 100 μL of sample (from the protein precipitation steps above) or of the water based calibration solution is pipetted.
b) 50 μL of the combined Griess reagent mixture is added with the 8-channel multiple pipette.
c) Then, for the measurement of nitrite, 100 μL of 1 M HCl is added with the 8-channel multiple pipette.

For the measurement of nitrite alone, the plate is allowed to stand at room temperature for 15 min before insertion in the plate reader for absorbance measurement.

Absorbance measurements are analogically transferred to the results collection sheet and input to the calculation spreadsheet.

Plate B: measurement of nitrite+nitrate (total Nitrogen oxides)

	1	2	3	4	5	6	7	8	9	10	11	12
**A**	○0	○0	○1	○2	○3	○4	○5	○6	○7	○8	○9	○10
**B**	○5	○5	○11	○12	○13	○14	○15	○16	○17	○18	○19	○20
**C**	○10	○10	○21	○22	○23	○24	○25	○26	○27	○28	○29	○30
**D**	○20	○20	○31	○32	○33	○34	○35	○36	○37	○38	○39	○40
**E**	○50	○50	○41	○42	○43	○44	○45	○46	○47	○48	○49	○50
**F**	○100	○100	○51	○52	○53	○54	○55	○56	○57	○58	○59	○60
**G**	○200	○200	○61	○62	○63	○64	○65	○66	○67	○68	○69	○70
**H**	○v	○v	○71	○72	○73	○74	○75	○76	○77	○78	○79	○80

Legend.

○0-200: NO_3^-calibrator (μM)	○n: sample (1-80)	

In the 96-well plate, for each well, the following volumes of sample and reagents are dispensed.

a) 100 μL of sample (from the protein precipitation steps above) or of the water based calibration solution is pipetted.

b) 50 μL of the combined Griess reagent mixture is added with the 8-channel multiple pipette.
c) Then, for the measurement of nitrite+nitrate, 100 μL of Vanadium(II) chloride (25 mM in 1 M HCl) solution is added with the 8-channel multiple pipette.
d) the plate is incubated at 50°C for 60 min, covered with its lid, then allowed to cool at room temperature for 5 min.
e) The plate is then inserted into the reader for the spectrophotometric measurement

Absorbance measurements are analogically transferred to the results collection sheet and input to the calculation spreadsheet.

Operative checklist for the measurement of nitrite and nitrate in red blood cells.

Protein precipitation

Procedure for red blood cells. To each thawed sample tube containing approx. 50 µL of separated RBCs, 100 microL of chilled sterile water is added, the tube stoppered and mixed by inversion to completely hemolyze and resuspend the contents.

A 10- µL subsample is withdrawn and diluted 1:50 for immediate measurement of hemoglobin concentration (see below, **plate C**).

A 100- µL subsample is dispensed in a 1.5-mL Eppendorf tube, followed by 200 µL of chilled acetonitrile. Each lot of 6 stoppered tubes is immediately centrifuged at at 12,000 rpm for 10 min and two 100- µL subsamples are withdrawn from each for loading in **plates A and B** for separate nitrite and nitrate measurement.

Sample plate preparation and measurement

According to the number of measured samples, one plate per measurement is prepared, with samples in matching positions. The sample loading scheme below is presented for the analysis of a 80-sample batch in 96-well plates.

Fresh reagent batches were prepared from the solids and used immediately after.

Plate A: measurement of nitrite

	1	2	3	4	5	6	7	8	9	10	11	12
**A**	○0	○0	○1	○2	○3	○4	○5	○6	○7	○8	○9	○10
**B**	○5	○5	○11	○12	○13	○14	○15	○16	○17	○18	○19	○20
**C**	○10	○10	○21	○22	○23	○24	○25	○26	○27	○28	○29	○30
**D**	○20	○20	○31	○32	○33	○34	○35	○36	○37	○38	○39	○40
**E**	○50	○50	○41	○42	○43	○44	○45	○46	○47	○48	○49	○50
**F**	○100	○100	○51	○52	○53	○54	○55	○56	○57	○58	○59	○60
**G**	○200	○200	○61	○62	○63	○64	○65	○66	○67	○68	○69	○70
**H**	○v	○v	○71	○72	○73	○74	○75	○76	○77	○78	○79	○80

Legend.

○0-200: NO_2^-calibrator (µM)	○n: sample (1-80)	

In the 96-well plate, for each well, the following volumes of sample and reagents are dispensed.

a) 100 µL of sample (from the protein precipitation steps above) or of the water based calibration solution is pipetted.
b) 50 µL of the combined Griess reagent mixture is added with the 8-channel multiple pipette.
c) Then, for the measurement of nitrite, 100 µL of 1 M HCl is added with the 8-channel multiple pipette.
d) For the measurement of nitrite alone, the plate is allowed to stand at room temperature for 15 min before insertion in the plate reader for absorbance measurement at 540 nm (xx-yy nm bandpass filter).

Absorbance measurements are analogically transferred to the results collection sheet and input to the calculation spreadsheet.

Plate B: measurement of nitrite

	1	2	3	4	5	6	7	8	9	10	11	12
**A**	○0	○0	○1	○2	○3	○4	○5	○6	○7	○8	○9	○10
**B**	○5	○5	○11	○12	○13	○14	○15	○16	○17	○18	○19	○20
**C**	○10	○10	○21	○22	○23	○24	○25	○26	○27	○28	○29	○30
**D**	○20	○20	○31	○32	○33	○34	○35	○36	○37	○38	○39	○40
**E**	○50	○50	○41	○42	○43	○44	○45	○46	○47	○48	○49	○50
**F**	○100	○100	○51	○52	○53	○54	○55	○56	○57	○58	○59	○60
**G**	○200	○200	○61	○62	○63	○64	○65	○66	○67	○68	○69	○70
**H**	○v	○v	○71	○72	○73	○74	○75	○76	○77	○78	○79	○80

Legend.

○0-200: NO_3^-calibrator (µM)	○n: sample (1-80)	

In the 96-well plate, for each well, the following volumes of sample and reagents are dispensed.

a) 100 µL of sample (from the protein precipitation steps above) or of the water based calibration solution is pipetted.
b) 50 µL of the combined Griess reagent mixture is added with the 8-channel multiple pipette.
c) Then, for the measurement of nitrite+nitrate, 100 µL of Vanadium(II) chloride (25 mM in 1 M HCl) solution is added with the 8-channel multiple pipette.
d) the plate is incubated at 50°C for 60 min, covered with its lid, then allowed to cool at room temperature for 5 min.
e) The plate is then inserted into the reader for spectrophotometric measurement at 540 nm (550 nm filter bandpass 10-14 nm).

Absorbance measurements are analogically transferred to the results collection sheet and input to the calculation spreadsheet.

Plate C: measurement of hemoglobin

	**1**	**2**	**3**	**4**	**5**	**6**	**7**	**8**	**9**	**10**	**11**	**12**
**A**	○5	○v	○1	○2	○3	○4	○5	○6	○7	○8	○9	○10
**B**	○5	○v	○11	○12	○13	○14	○15	○16	○17	○18	○19	○20
**C**	○10	○v	○21	○22	○23	○24	○25	○26	○27	○28	○29	○30
**D**	○10	○v	○31	○32	○33	○34	○35	○36	○37	○38	○39	○40
**E**	○20	○v	○41	○42	○43	○44	○45	○46	○47	○48	○49	○50
**F**	○20	○v	○51	○52	○53	○54	○55	○56	○57	○58	○59	○60
**G**	○40	○v	○61	○62	○63	○64	○65	○66	○67	○68	○69	○70
**H**	○40	○v	○71	○72	○73	○74	○75	○76	○77	○78	○79	○80

Legend.

○5-40: Hb calibrator (µM)	○n: sample (1-80)	

In the 96-well plate, for each well, the following volumes of sample and reagents are dispensed.

a) 200 µL of water based hemoglobin calibration solution is pipetted (2 x 40-20-10-5 microM) in wells 1A-H.
b) 200 µL of sample in wells 3A-12H
c) The plate is then inserted into the reader for spectrophotometric measurement at 450 nm (filter bandpass 8.5-16 nm).

Absorbance measurements are analogically transferred to the results collection sheet and input to the calculation spreadsheet.

Hemocytometric characterization of blood samples

Total hemoglobin concentration (mg/dL) and hematocrit fraction (%) are used to normalize analytically measured concentrations of nitrite and nitrate in the hemolyzates to actual concentrations in the red blod cells.

For the hemocytometric characterization of each fresh blood sample, use the 150-m µL whole blood sample that was prepared as step (c) of the whole blood fractionation procedure. For field studies, this measurement is performed on as fresh as possible whole blood, if similar equipment is available at the site of sample collection.

That shown is a typical report with indicative values of standard deviation and relative standard deviation for a quintuplicate measurement of the sample Results from the results collection sheet are input to the calculation spreadsheet

MW(hHB)=64.454	**Name**	**Subj_ID**	**SD**	**CV**
day	**Date**	25/05/2021		
10^6_n/uL	**WBC**	9,0	0,2	2,2
10^6_n/uL	**LYM**	2,2	0,1	3,8
10^6_n/uL	**GRAN**	5,7	0,1	2,3
g/dL_WB	**HGB**	15,2	0,8	5,2
g/dL_RBC	**MCH**	33,4	0,3	0,8
HbcorrHt_g/dL	**MCHC**	33,8	0,3	1,0
10^6_n/uL	**RBC**	4,5	0,3	5,6
fL=10^-12 L	**MCV**	98,9	0,4	0,4
% v/v	**HCT**	44,9	2,7	5,9
%	**RDW%**	14,9	0,1	0,9
10^6_n/uL	**PLT**	261,2	10,5	4,0
10^6_n/uL	**MPV**	8,4	0,1	1,0
HbcorrHt_g/dL	HGB/HCT	33,7	7,9	23%
HbcorrHt_mM		5,2	1,2	23%

Calculation and data analysis

Nitrite and nitrate concentration.

A 14-point, 7-level calibration curve is calculated by least-squares linear regression of absorbance *vs.* concentration data obtained from the standard solutions of each reading plate. One step of calibrator iteration is performed when more than one calibrator shows an accuracy higher than ± 5%.

Typical absorbance-concentration relationship for nitrite is reported below.

n_dat	[ANA] nom (uM)	[ANA] ite (uM)	ABS	ANA ricalc.	±DS	Accuracy (%)	Precision (%)	Regression output	
0	***0,0***	0,0		***-0,25***					
1	0	0	0,003	**0,47**	**0,65**			intc =	0,001
2	0	0	0,005	**0,96**	**0,65**			err_intc =	0,001
3	5	5,2	0,022	**5,08**	**0,65**	-5,5%	15,2	slope =	0,00412
4	5	5,2	0,023	**5,33**	**0,65**	-0,8%	14,5	err_slope =	0,000
5	10	9,9	0,041	**9,69**	**0,65**	-4,0%	7,9	Corr_coef_r2 =	1,0000
6	10	9,9	0,043	**10,18**	**0,65**	0,9%	7,5	n_dat	14
7	20	20,0	0,082	**19,64**	**0,65**	-2,5%	3,9	Y(LoD) =	0,007
8	20	20,0	0,085	**20,37**	**0,65**	1,1%	3,7	LoD (3SD) (uM) =	**1,35**
9	50	48,0	0,196	**47,29**	**0,68**	-1,7%	1,7		
10	50	48,0	0,202	**48,75**	**0,62**	1,3%	1,5		
11	100	101,3	0,416	**100,65**	**0,65**	-0,6%	0,7		
12	100	101,3	0,421	**101,87**	**0,65**	0,6%	0,7		
13	200	199,9	0,826	**200,11**	**0,65**	0,2%	0,4		
14	200	199,9	0,824	**199,62**	**0,65**	-0,1%	0,4		

The spreadsheet calculates for each sample the concentration corresponding to its absorbance reading and its associated standard error.

Plate_ID	Samp_ID	ABS	ANA (uM)	err.std uM
B10	Samp_ID	0,553	133,9	1,0

Hemoglobin concentration in the hemolizate.

A 8-point, 4-level calibration curve is calculated by least-squares linear regression of absorbance *vs.* concentration data obtained from the hemoglobin standard solutions. One step of calibrator iteration is performed when more than one calibrator shows an accuracy higher than ± 5%. Typical absorbance-concentration relationship is reported below.

n_dat	[ANA] nom (uM)	[ANA] ite (uM)	ABS	ANA ricalc.	±DS	Accuracy (%)	Precision (%)	Regression output	
0	***0,0***	0,0		***-0,25***					
1	2,50	2,3	0,090	2,34	0,13	0,07%	5,5	intc =	0,022
2	2,50	2,3	0,090	2,34	0,13	-0,07%	5,5	err_intc =	0,001
3	5,00	5,0	0,165	4,93	0,13	-0,94%	2,6	slope =	0,02912
4	5,00	5,0	0,168	5,02	0,13	0,94%	2,6	err_slope =	0,000
5	10,00	9,4	0,302	9,64	0,21	2,22%	2,2	Corr_coef_r2 =	0,9999
6	10,00	9,4	0,290	9,22	0,09	-2%	1,0	n_dat	14
7	20,00	20,3	0,613	20,30	0,13	0,24%	0,6	Y(LoD) =	0,029
8	20,00	20,3	0,610	20,21	0,13	-0,24%	0,6	LoD (3SD) (uM) =	**0,27**

The spreadsheet calculates for each sample the concentration corresponding to its absorbance reading and its associated standard error.

Plate_ID	Samp_ID	ABS	ANA (uM)	err.std uM
B10	Samp_ID	0,731	24,4	0,2

Calculation of the extended error of determination

a) For the determination of extended uncertainty of nitrate concentration in the sample from the separate measurement of nitrite and total nitrogen oxides (NOx), equation (1) is used, where the Standard Deviation (SD) of the measurements are employed.

$$SD(NO_3) = (SD(NO_2)^2 + SD(NOx)^2)^{1/2} \text{ (equation 1)}$$

	[NO2]			[NOx]			[NO3]		
**SampID**	**uM**	**SE**	**CV%**	**uM**	**SE**	**CV%**	**uM**	**SE**	**CV%**
SampID	**2,85**	**0,9**	**33%**	**11,11**	**2,6**	**24%**	**11.11 – 2.85 = 8,26**	**$(0.9^2 + 2.6^2)^{½}$ = 2,78**	**2.78 / 8.26 = 33%**

b) For the determination of extended uncertainty of concentrations normalized to hemolizate hemoglobin concentration, equation (2) is used, where the Coefficient-of-Variation (100*SD%) of the measurements are employed.

The four-step calculation is highlighted in the tabulated example.

$$CV(NO_2)/Hb = (CV(NO_2)^2 + CV(Hb)^2)^{1/2} \text{ (equation 2)}$$

The four-step calculation is highlighted in the tabulated example.

**SampID**	[Hb]			$[NO_2]$			$[NO_x]$			$[NO_3]$		
	**mM**	**SE**	**CV%**	**uM**	**SE**	**CV%**	**uM**	**SE**	**CV%**	**uM**	**SE**	**CV%**
**In hemolyzate**	2,42	0,05	2,1	2,85	0,90	31,6	11,11	2,60	23,4	8,26	2,75	33,3
**In RBC**	5,20	0,05	1,0									
**uM/mM Hb**				1,18	0,37	31,6	4,59	1,08	23,5	3,41	1,14	33,4
**uM in RBC**				6,12	1,94	31,7	23,87	5,61	23,5	17,75	5,93	33,4
